# Varus placement of the tibial component reduces the potential risk of fracture with adequate bony coverage in the Oxford unicompartmental knee arthroplasty

**DOI:** 10.1038/s41598-023-48659-4

**Published:** 2024-01-13

**Authors:** Tomoyuki Kamenaga, Takafumi Hiranaka, Yoshihito Suda, Takaaki Fujishiro, Koji Okamoto, Ryosuke Kuroda, Tomoyuki Matsumoto

**Affiliations:** 1 Department of Orthopedic Surgery and Joint Surgery Center, Takatsuki General Hospital, 1-3-13, Kosobe-Cho, Takatsuki, Osaka 569-1192 Japan; 2 Department of Orthopedic Surgery, Kobe University Graduate School of Medicine, Kusunoki-Cho, Chuo-Ku, Kobe, Hyogo 650-0017 Japan

**Keywords:** Anatomy, Medical research

## Abstract

A short keel-cortex distance (KCD), especially to the posterior cortex, is a potential risk factor for tibial plateau fracture after Oxford mobile-bearing unicompartmental knee arthroplasty (OUKA). This study aimed to evaluate the effect of tibial component alignment in the coronal plane and tibial proximal morphology on the KCD. Included in this study were 51 patients scheduled for primary Oxford medial unicompartmental knee arthroplasty (UKA). The anterior and posterior KCD were preoperatively assessed using 3D simulation software with the component set perpendicular to the tibial mechanical axis (neutral), 3° valgus, 3° varus, and 6° varus, relative to neutral alignment. We evaluated the existence of overhanging medial tibial condyle where the medial eminence line, the line including the medial tibial eminence parallel to the tibial axis, passes outside of the tibial shaft. In all component alignments, patients with a medial overhanging condyle had significantly shorter posterior KCD than those without. In patients with a medial overhanging condyle, the posterior KCD significantly increased when the tibial component was placed in 3° varus (4.6 ± 1.5 mm, *P* = 0.003 vs neutral, *P* < 0.001 vs 3° valgus) and 6° varus (5.0 ± 1.4 mm*, P* < 0.001 vs neutral, *P* < 0.001 vs 3° valgus) compared with in neutral (3.5 ± 1.9 mm) or 3° valgus (2.8 ± 1.8 mm). In OUKA, varus implantation increased the KCD. This could potentially decrease the risk of fracture, even in knees with the overhanging medial condyle. Conversely, valgus implantation of the tibial component shortened the KCD, and should therefore be avoided.

## Introduction

Cementless Oxford mobile-bearing unicompartmental knee arthroplasty (OUKA) reportedly achieves comparable clinical results compared with cemented OUKA, with markedly reduced radiolucent lines^1^. However, medial tibial plateau fracture is a serious complication after cementless OUKA. Its occurrence after OUKA has typically been attributed to technical errors^2–4^. The keel of the tibial component was shown to play an important role in fracture occurrence, and a shorter distance between the keel of the tibial component and the cortex (keel-cortex distance; KCD) was associated with an increased risk of fracture^5^.

Fractures are more common in Asian countries (3.8–8.0%)^6–9^ than in non-Asian countries (< 1%)^10–12^, perhaps due to the high prevalence of constitutional varus^13^ in Asian patients^14^. Patients with proximal tibial vara also have high prevalence of fractures^8,9^. The KCD was recently reported to be shorter in patients with proximal tibial vara^5^. Varus placement has been commonly implemented in fixed-bearing UKA; it can avoid the stress concentration and potentially prevent failure^15,16^. Slight varus placement of the tibial component could therefore be an effective procedure in OUKA and might widen the KCD, thus decreasing the risk of fractures. However, the effect of varus placement on the KCD has not yet been evaluated. This simulation study uses 3D-CT to evaluate KCDs in relation to the tibial component varus/valgus alignment. We hypothesised that the KCD is longer when the tibial component is in varus placement compared with perpendicular or valgus alignment, even in the proximal tibial vara.

## Materials and methods

This research has been approved by the institutional review board of Takatsuki general hospital (No. 2020-14). All methods were carried out in accordance with relevant guidelines and regulations. We studied 51 unilateral lower limbs in 51 consecutive patients that underwent primary OUKA in our hospital between February and April 2020. There were 39 women and 12 men (mean age 71.7 ± 6.9 years, mean body mass index 25.3 ± 3.6 kg/m^2^, HKA 7.9° ± 5.5° in varus). As this is an observational study without patient invasion or intervention, we did not obtain consent directly from each patient. The need for informed consent was waived by the ethics committee of Takatsuki general hospital. However, we disclosed the purpose of the study and information as an opt-out, and guaranteed the opportunity to refuse participation in accordance with the ethic committee in Takatsuki general hospital. All were diagnosed with anteromedial osteoarthritis^17^ and selected according to previously described guidelines^18^. Flexion contracture of the knee was < 15°, and the HKA angle was < 15° in all knees.

### Measurement of the keel-cortex distances and tibial component coverage

**Figure 1:**
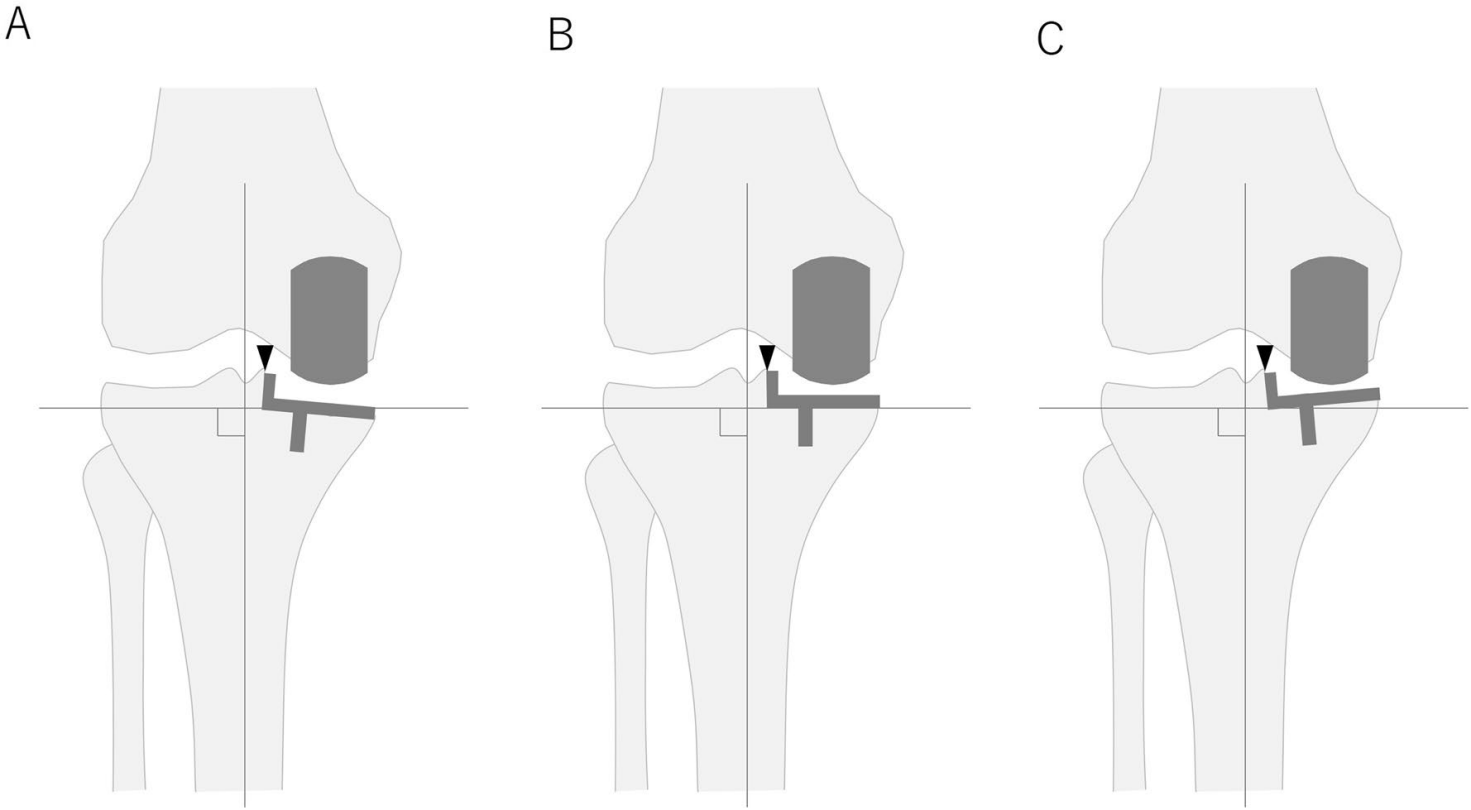
Images showing the setting of the tibial component coronal alignment. The tibial component neutral position was set perpendicular to the tibial mechanical axis (**A**). The tibial component varus (**B**)/valgus (**C**) alignment was adjusted with the tip of medial intercondylar eminence.

**Figure 2:**
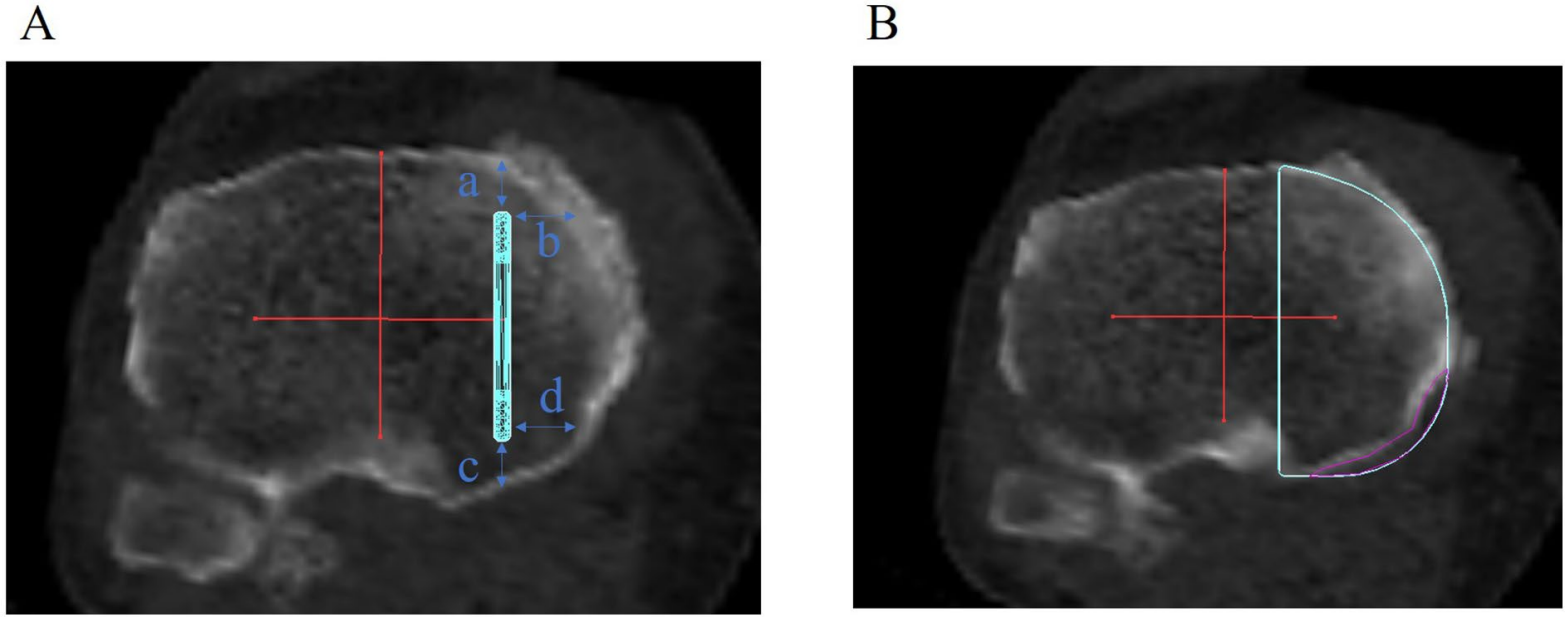
(**A**) The distance between the keel and tibial cortex was measured along the axial plane in various regions: anterior (a), anteromedial (b), posterior (c), and posteromedial (d). (**B**) Amount of over-coverage was measured by calculating area within the enclosed line.

As the routine examination, whole-leg CT scans were performed in every patient with 2 mm thick slices using Aquilion ONE (Toshiba, Tokyo, Japan). Patients were positioned on the table in a supine position. Scanning were performed from the hip to the ankle joint with the patient in a knee-extension with the patella facing upward. The obtained image datasets were imported into 3D multiplanar reconstruction image simulation software (ATHENA; Soft Cube, Osaka, Japan). This system has computer-aided design (CAD) data of various implants, including OUKA, and can accurately assess the implant position relative to the bone landmark in TKA^19^. The tibial mechanical axis (TMA) passed through the centre of the medial and lateral tibial eminences and the centre of the talar dome. The tibial AP line connects the middle of the PCL and the medial border of the patellar tendon attachment to the tibial tubercle, as described previously^20^. The proximal tibial articular surface was cut perpendicular to the TMA with a posterior inclination of 7° and 4 mm below the medial joint lines. The cutting line at the articular surface was determined to be parallel with the tibial AP line through the tip of medial intercondylar eminence, which is accessible in the small operating field in medial UKA. Next, KCD and over-coverage of the tibial component were evaluated when the component was set perpendicular (neutral), 3° valgus (valgus3), 3° varus (varus3), and 6° varus (varus6) to the TMA. The rotational centre of the tibial component varus/valgus alignment was set at the tip of medial intercondylar eminence (Fig. 1). Four KCDs were measured; anterior, anteromedial, posterior and posteromedial (Fig. 2A). Oxford partial knee component size (Zimmer Biomet, Warsaw, IN) was selected based on the medio-lateral dimension of the tibial cutting surface so that medial edge of the component was flush, but never with undercover of the medial tibial cortex. Under- and overhang of the anterior part within 3 mm was tolerated. The amount of over-coverage was measured by calculating the area within the enclosed line, as shown in Fig. 2B. Osteophytes were excluded from the measurement range.

### Measurement of the tibial morphology

**Figure 3:**
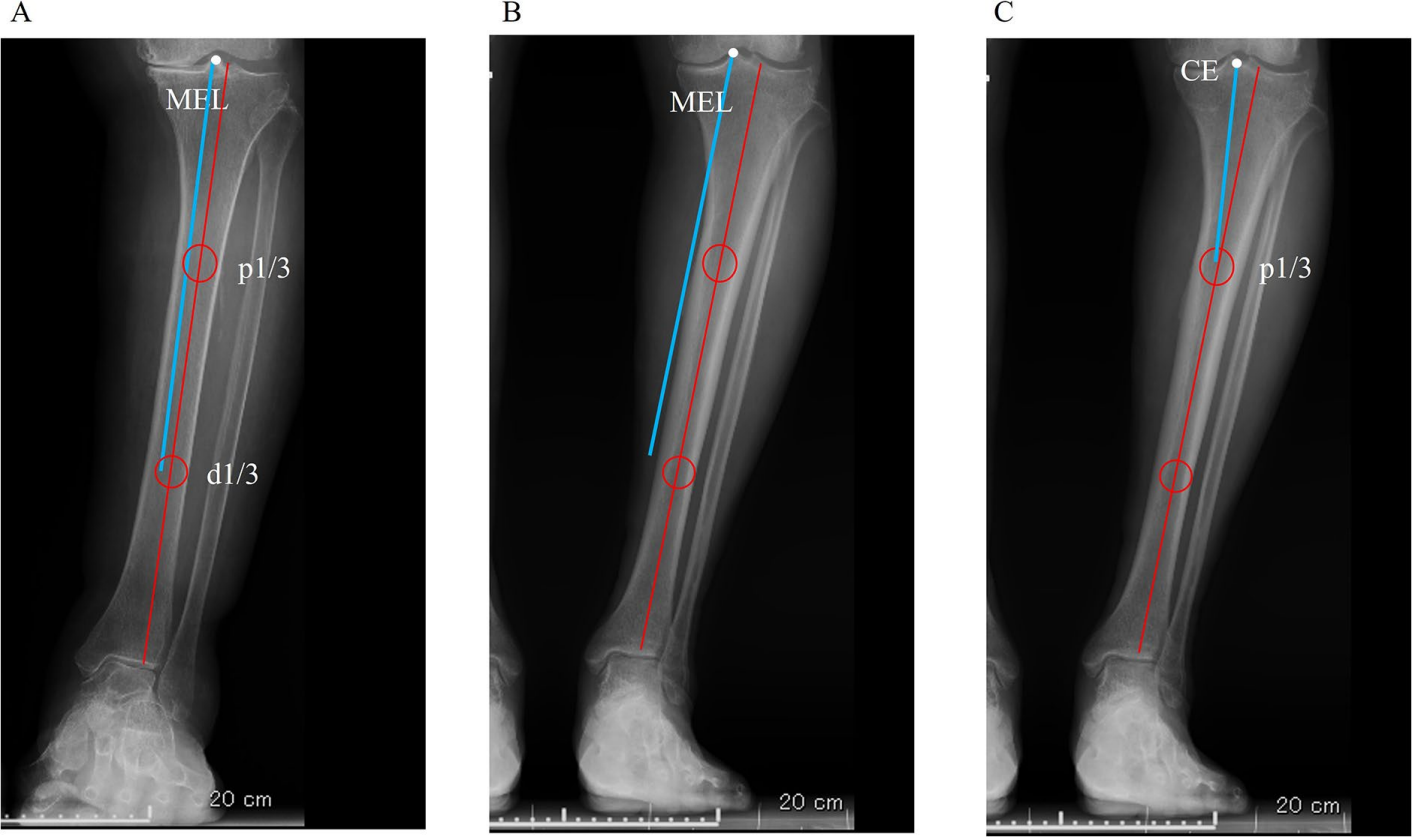
(**A**) Intramedullary type in the medial eminence line (MEL) classification. (**B**) Extramedullary type in the MEL classification. (**C**) Measurement of proximal tibia vara angle.

All preoperative weight-bearing radiographs were obtained one month before surgery according to a previously reported standardised protocol^21^. Briefly, the patella was placed forward, with the ankle in the neutral position. Patients were instructed to stand upright with extended knees with both heels and hallux in contact with the floor. Tibial morphology was assessed with the medial eminence line (MEL), as previously described^8,9^. The MEL was drawn passing through the apex of the medial intercondylar eminence and parallel to the tibial anatomical axis (TAA). The TAA was defined as a line connecting the centres of the proximal 1/3 (p1/3) and distal 1/3 (d1/3) of the tibia^22^. If the MEL passed lateral to the medial cortex of the tibia, the tibia was classified as ‘intramedullary’, and the medial condyle was considered to be normal shape (Fig. 3A). Otherwise, if the MEL passed medial to the medial cortex, it was classified as ‘extramedullary’, and the medial condyle was considered to be very overhanging (Fig. 3B)^9^. In addition, the proximal tibia vara angle (PVA) was evaluated to assess medial bowing in the proximal tibia with the AP radiographs of the lower extremity, according to the previous article^22^. The PVA was defined as the angle between the TAA and the line connecting the centre of the tibial eminence (CE) and the midpoint of the proximal 1/3 of the tibia (Fig. 3C).

### Statistical analysis

Intraclass and interclass correlation coefficients (CC) were calculated to examine the reproducibility of the measurements. All measurements were performed twice by one surgeon and once by another examiner. CCs for intra- and inter-observer reliability were > 0.81 (range 0.81–0.96) for all measurements (Table 1).

**Table 1:** Reliability of all measurements when the component was set in neutral position.

	ICC Intra-rater (95% CI)	ICC Inter-rater (95% CI)
Anterior KCD, mm	0.86 (0.63–0.98)	0.96 (0.89–0.99)
Anteromedial KCD, mm	0.82 (0.50–0.97)	0.89 (0.58–0.97)
Posterior KCD, mm	0.88 (0.62–0.98)	0.87 (0.60–0.99)
Posteromedial KCD, mm	0.90 (0.80–0.97)	0.83 (0.54–0.97)
Over-coverage, mm^2^	0.82 (0.55–0.96)	0.86 (0.61–0.97)

*KCD* keel-cortex distance, *ICC* intraclass correlation coefficient, *CI* confidence interval.

All values are reported as mean ± standard deviation (SD). Results were analysed using StatView 5.0 (Abacus Concepts Inc., Berkeley, CA, USA). All parameters were normally distributed. Spearman’s rank correlation analysis was used to assess the correlation of PVA with the KCDs and amount of over-coverage. The KCDs in each region and amount of over-coverage were compared between two groups (extramedullary and intramedullary) using unpaired t-tests. They were compared using repeated-measures ANOVA with within-factors (neutral, valgus3, varus3, varus6) in both groups (extramedullary and intramedullary) using Bonferroni correction. Additionally, to investigate the benefit of varus placement over neutral placement, the difference in KCDs between varus placement (varus3 and 6) and neutral placement were compared between extramedullary and intramedullary groups using unpaired t-tests.

Post-hoc power analysis was performed using G*Power 3^23^. For repeated measures ANOVA with within-factors, the study is expected to provide the power (1 − β) of 0.99 and 0.81 for detecting an effect size (f) of 0.3 with type-I error (α) of 0.05, in intramedullary (n = 34) and extramedullary groups (n = 17), respectively. For unpaired t-tests, the effect size was calculated using means and SDs based on the Hedges’ g for each parameter and a 95% confidence interval (CI) for effect sizes^24^.

### Ethical approval

This research has been approved by the IRB of the authors’ affiliated institutions. (2020-14).

## Results

For all subjects, significantly shorter KCDs and larger over-coverage in valgus3 were found compared with the others (neutral, varus3, and varus6) (*P* < 0.0083 after Bonferroni correction; Table 1). Posterior KCDs showed lower values in neutral compared with varus3 and varus6 (*P* < 0.0083 after Bonferroni correction; Table 2).

**Table 2:** Keel-cortex distance and amount of over-coverage.

	Valgus 3	Neutral	Varus 3	Varus 6	*P*-value
Anterior KCD, mm	6.1 (1.9)*	6.8 (1.8)	7.1 (1.8)	7.5 (1.8)	< 0.001
Anteromedial KCD, mm	7.8 (1.8)*	8.4 (1.9)	8.7 (2.3)	9.2 (2.2)	< 0.001
Posterior KCD, mm	3.8 (2.2)*	4.6 (2.1) **	5.5 (1.8)	5.8 (1.7)	< 0.001
Posteromedial KCD, mm	8.5 (1.8)*	9.4 (1.9)	10.1 (2.3)	10.5 (2.2)	< 0.001
Over-coverage, mm^2^	24.9 (29.5)*	9.6 (16.2)	4.9 (13.7)	8.1 (11.2)	< 0.001

Date presented as mean (standard deviation).

*KCD* keel-cortex distance.

*Significant difference with Bonferroni correction (*P* < 0.0083) vs. Neutral, Varus 3, and Varus 6.

**Significant difference with Bonferroni correction (*P* < 0.0083); vs. Varus 3 and Varus 6.

There were 34 patients (67%) in the intramedullary group and 17 patients (33%) in the extramedullary group. No statistically significant differences were noted in terms of age, sex, BMI, preoperative coronal alignment, and maximum flexion angle (Table 3). However, there was significantly higher PVA in the extramedullary group than in the intramedullary group (6.8 ± 2.8° vs. 3.1 ± 4.3°, *P* < 0.001, Hedges' g = 0.94, 95% CI 0.41 to 1.56).

**Table 3:** Preoperative demographic data.

	Intramedullary	Extramedullary	*P*-value
Number of cases	34	17	
Sex (female/male)	26/8	13/4	1.00
Age (years)	72.4 ± 7.5 (52–88)	73.2 ± 7.0 (56–90)	0.84
Coronal alignment in varus (°)	7.7 ± 5.4 (0.5–15.2)	8.1 ± 5.7 (0.2–17.2)	0.77
Maximum knee flexion (°)	125.4 ± 11.5 (100–150)	128.8 ± 12.4 (95–150)	0.50

Date presented as mean ± standard deviation (range).

### Correlations between PVA with KCDs and over-coverage

PVA showed significant negative correlations with posterior and posteromedial KCDs for all within-factors. High correlations were found between PVA and the amount of over-coverage in a neutral position (Table 4).

**Table 4:** Correlation between the proximal vara angle and the keel-cortex distances in each region.

	Proximal vara angle	*P* value
Anterior-KCD, mm		
Valgus3	− 0.03	0.81
Neutral	0.24	0.09
Varus3	− 0.19	0.18
Varus6	0.08	0.59
Anteromedial-KCD, mm		
Valgus3	− 0.09	0.54
Neutral	− 0.11	0.45
Varus3	− 0.12	0.40
Varus6	− 0.20	0.17
Posterior-KCD, mm		
Valgus3	− 0.55*	< 0.001
Neutral	− 0.71*	< 0.001
Varus3	− 0.64*	< 0.001
Varus6	− 0.61*	< 0.001
Posteromedial-KCD, mm		
Valgus3	− 0.66*	< 0.001
Neutral	− 0.63*	< 0.001
Varus3	− 0.55*	< 0.001
Varus6	− 0.54*	< 0.001
Over-coverage		
Valgus3	0.23	0.11
Neutral	0.58*	< 0.001
Varus3	0.22	0.12
Varus6	0.08	0.56

*KCD* keel-cortex distance.

*Statistically significant correlation (*P* < 0.05).

### Comparison of KCDs and over-coverage between extramedullary and intramedullary groups

Comparison between the groups is shown in Table 5. The anterior and anteromedial KCDs showed no significant difference between the groups for all within-factors. However, the posterior and posteromedial KCDs were significantly lower in the extramedullary group than in the intramedullary group for all within-factors (valgus3, neutral, varus3, and varus6). The amount of over-coverage was significantly larger in the extramedullary group than in the intramedullary group when the tibial component was set in a neutral position.

**Table 5:** Comparison of keel-cortex distances and over-coverage using medial eminence line classification.

	Extramedullary	Intramedullary	*P* value	Hedge’s g	95%CI	
Anterior-KCD, mm						
Valgus3	6.0 (1.4)	6.1 (2.1)	0.80	− 0.05	− 0.63	0.53
Neutral	6.7 (1.6)	6.9 (2.0)	0.78	− 0.10	− 0.69	0.48
Varus3	7.1 (1.3)	7.2 (1.9)	0.83	− 0.06	− 0.64	0.52
Varus6	7.2 (1.4)	7.6 (2.0)	0.50	− 0.22	− 0.80	0.37
Anteromedial-KCD, mm						
Valgus3	7.6 (1.4)	7.9 (1.9)	0.61	− 0.30	− 0.90	0.28
Neutral	7.8 (2.0)	8.7 (1.9)	0.13	− 0.46	− 1.05	0.13
Varus3	8.1 (1.3)	8.9 (2.0)	0.25	− 0.43	− 1.03	0.15
Varus6	8.5 (1.3)	9.4 (2.4)	0.09	− 0.47	− 1.06	0.12
Posterior-KCD, mm						
Valgus3	2.8 (1.8)	4.2 (2.2)	0.03*	− 0.67	− 1.27	− 0.07
Neutral	3.5 (1.9)	5.2 (2.2)	0.009*	− 0.79	− 1.41	− 0.20
Varus3	4.6 (1.5)	5.9 (1.8)	0.02*	− 0.75	− 1.36	− 0.16
Varus6	5.0 (1.4)	6.2 (1.8)	0.01*	− 0.70	− 1.31	− 0.12
Posteromedial-KCD, mm						
Valgus3	7.4 (1.9)	9.2 (2.3)	0.006*	− 0.81	− 1.43	− 0.22
Neutral	8.1 (1.9)	10.2 (2.1)	0.003*	− 1.02	− 1.64	− 0.41
Varus3	9.1 (1.9)	10.6 (2.2)	0.01*	− 0.70	− 1.31	− 0.11
Varus6	9.8 (1.8)	10.9 (2.3)	0.04*	− 0.60	− 1.20	− 0.01
Over-coverage, mm^2^						
Valgus3	30.8 (32.2)	22.0 (28.1)	0.32	0.30	− 0.29	0.88
Neutral	20.7 (17.2)	4.6 (13.1)	0.005*	1.09	0.48	1.72
Varus3	4.8 (9.0)	5.0 (12.2)	0.64	− 0.03	0.61	0.56
Varus6	5.8 (9.5)	9.3 (11.8)	0.30	− 0.31	− 0.90	0.27

Date presented as mean (standard deviation).

*KCD* keel-cortex distance.

*Statistically significant difference (*P* < 0.05).

### Comparison of KCDs and over-coverage within-factors (valgus3, neutral, varus3, and varus6) in both extramedullary and intramedullary groups

**Figure 4:**
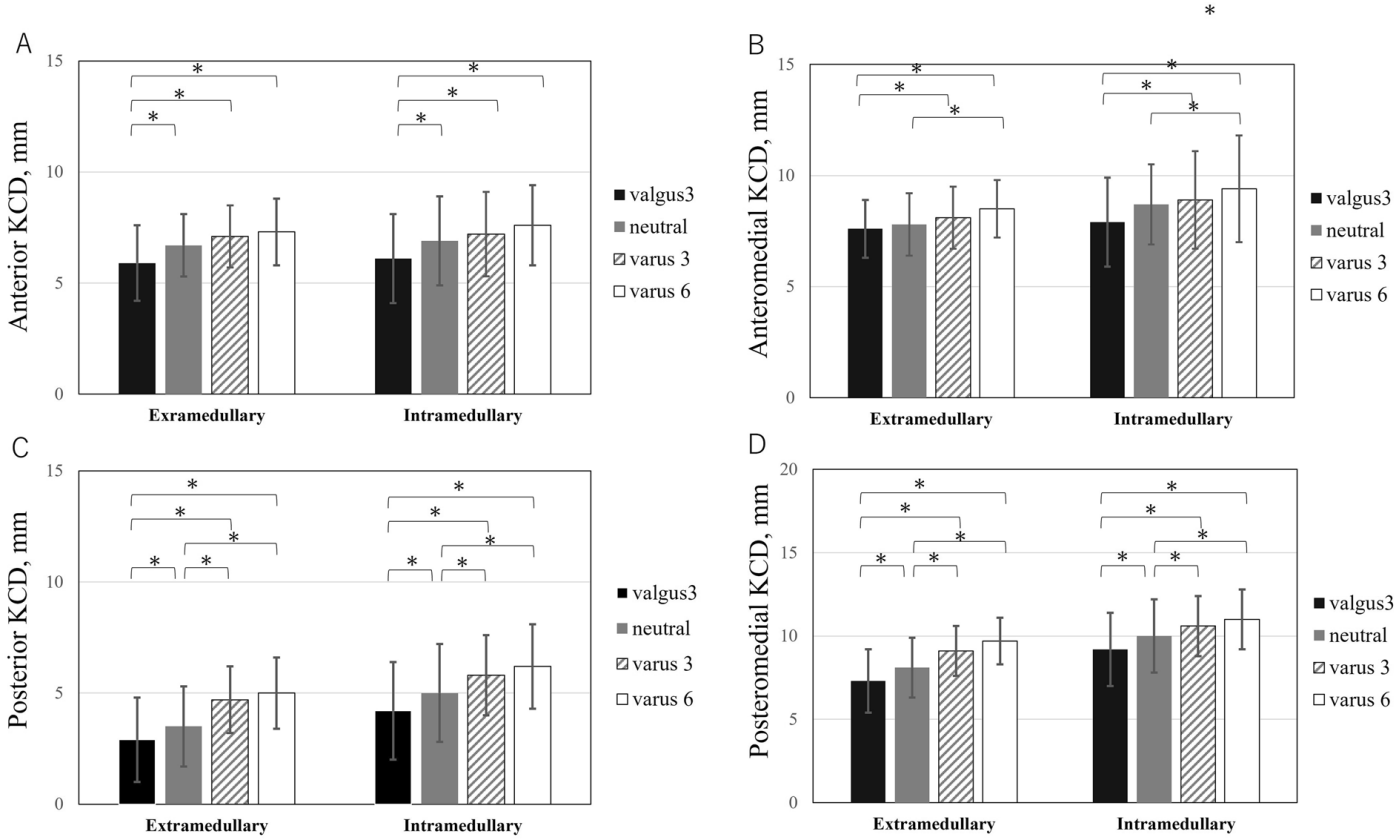
Comparison of KCDs in four regions (**A**. anterior **B**. anteromedial **C**. posterior **D**. posteromedial) within-factors (valgus3, neutral, varus3, and varus6) in both extramedullary and intramedullary groups. *Statistically significant difference (*P* < 0.05).

Significantly shorter KCDs in valgus3 was found compared with the others (neutral, varus3, and varus6) in both groups (*P* < 0.0083 after Bonferroni correction; Fig. 4). Posterior and posteromedial KCDs had lower values in neutral than in varus3 and varus6 (*P* < 0.0083 after Bonferroni correction; Fig. 4).

**Figure 5:**
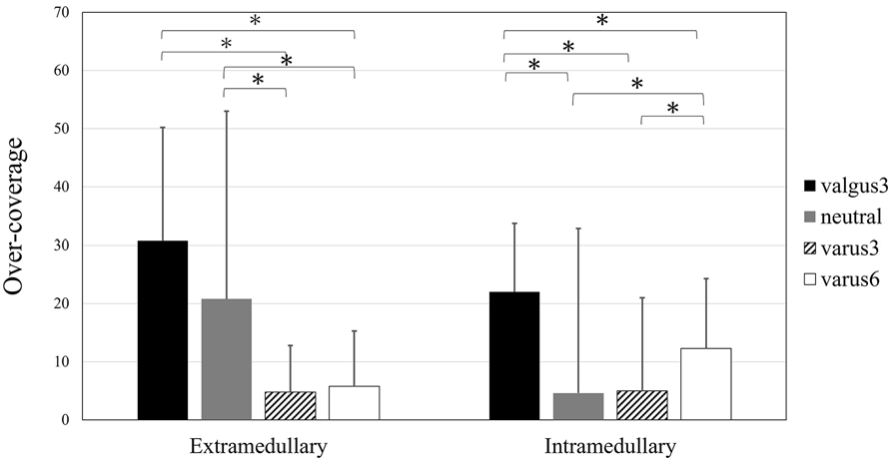
Comparison of over-coverage within-factors (valgus3, neutral, varus3, and varus6) in both extramedullary and intramedullary groups. *Statistically significant difference (*P* < 0.05).

Regarding the amount of over-coverage, in the intramedullary group, there was a significantly higher value in valgus3 than in the others (neutral, varus3, and varus6). In the extramedullary group there was a significantly higher value in valgus3 than in varus3 and varus6. Additionally, the amount of over-coverage was significantly higher in neutral than those in varus3 and varus6 (*P* < 0.0083 after Bonferroni correction; Fig. 5).

### Comparison of difference in KCDs from neutral to varus placement (varus3 and varus6) between extramedullary and intramedullary groups

Significantly larger differences were found in KCDs from neutral to varus3 and varus6 in extramedullary group compared with the intramedullary group (varus3 minus neutral; 1.15 ± 0.86 mm vs. 0.70 ± 0.65 mm, *P* = 0.03, Hedges' g = 0.62, 95% CI = 0.04 to 1.23. varus6 minus neutral; 1.54 ± 1.01 mm vs. 0.97 ± 0.88 mm, *P* = 0.04, Hedges' g = 0.61, 95% CI = 0.02 to 1.21).

## Discussion

In this 3D simulation study, the KCD was longer and the over-coverage was smaller in accordance with varus implantation. Meanwhile, a valgus implantation shortened the KCD and increased the amount of over-coverage. Based on these results, a varus implantation seems to be beneficial in maintaining sufficient KCD that might decrease the risk of fracture. These results confirmed our prior hypothesis. This is the first study to describe the effects of tibial component coronal alignment on KCDs and bony coverage for OUKA, which could be informative for surgeons in preoperative planning.

Posterior and posteromedial KCDs were shorter in patients with overhanging medial tibial plateaus, which is similar to the previous findings^5^. In addition, larger over-coverage was observed in patients with overhanging medial tibial plateau than those without. PVA showed significant correlation with the posterior and posteromedial KCDs. We also found higher PVAs in the extramedullary group than in the intramedullary group. Qualitative assessment using MEL classification thus reflects a proximal tibial vara and is a simple and useful means of predicting fractures. Overhanging medial tibial plateau reportedly has a higher risk of fracture^5,8,9^, so slight varus alignment of the tibial component is especially recommended in such knees. This information may be helpful when preparing the keel slot.

In both extramedullary and intramedullary groups, all KCDs were significantly lower when the tibial component was set in 3° valgus relative to the tibial AP axis than when set in a neutral position and 3° and 6° varus relative to it. There was a larger amount of over-coverage when the component was in valgus alignment than when it was set in neutral or varus alignment. This suggests that the valgus alignment of the tibial component decreases the bone mass supporting the tibial components and may be a risk factor for fractures in OUKA. Previous studies using the finite-element model demonstrated a significant increase of strain on the medial aspect of the proximal tibia following UKA in the setting of valgus implantation of tibial components^25,26^. Moreover, valgus implantation of the tibial component seems to cause deterioration of the coverage. Surgeons should therefore avoid the valgus implantation of the tibial component in OUKA.

Posterior and posteromedial KCDs were shorter in neutral than in 3° or 6° varus. In addition, increases in KCDs from neutral to 3° or 6° varus were significantly larger in the extramedullary group than in the intramedullary group. Regarding component coverage, the extramedullary group had significantly larger over-coverage than in the intramedullary group when the tibial component was set in a neutral position. Furthermore, in the extramedullary group, the amount of over-coverage was significantly larger in neutral alignment compared with 3° and 6° varus. Implantation in slight varus alignment seems to provide an advantage for the surgeon because of increased bony support under the tibial tray and achieving adequate component coverage, especially for patients with overhanging medial plateaus who are at high risk of posterior tibial cortical damage. The benefits of a slight varus alignment of the tibial component on joint line preservation, natural knee kinematics and better clinical outcomes have been reported^15,16,27^. The optimal target should therefore be slight varus alignment instead of placement perpendicular to the mechanical axis, especially in patients with medial overhanging tibias. However, the traditional extramedullary alignment resection guide was designed to cut perpendicular to the mechanical axis, so it is difficult to cut the proximal tibia accurately in a slight varus alignment without navigation or patient-specific instrumentation. Hiranaka et al. developed a new slidable fixator instead of the standard fixator to set the extramedullary rod on the leg. This enables an intentional varus tibial cut for OUKA^28^. This technique could be a simple and useful alternative means of obtaining an intentional varus tibial cut in OUKA.

This study has a number of limitations**.** First, due to the nature of this simulation study, actual postoperative cases were not examined. Such cases should be examined to seek the direct association of coronal alignment, shorter KCDs and postoperative fractures and to check that varus implantation is not a trade-off for inferior long-term implant survival. Nevertheless, the primary purpose of this study was to investigate the effect of the varus/valgus alignment of the tibial component on the KCD and bony coverage. A simulation study adjusts for confounders (component positions in sagittal and axial plane) influencing the KCD and bony coverage, which could not be adjusted for in actual postoperative cases. A second limitation of this study is that tibial component size is often chosen based on the AP diameter of the tibial cut surface, however in this study tibial component is chosen to minimize the medial side overhang, since the association of medial overhang with poor clinical outcome and postoperative pain have been reported^29^. This difference in size selection may lead to different results. Finally, our study population was limited to Japanese patients undergoing UKA. Differences in the shape of the tibia have been reported in differing ethnicities^30,31^. As tibial fracture is substantially common in Japan, however, the information might be important in Japanese patients and maybe in other Asian ethnicities with frequently reported overhanging medial tibial plateau.

## Conclusions

In OUKA, varus implantation increased the KCD and this may decrease the risk of fracture, even in knees with overhanging medial condyle. By contrast, the KCD is shortened by valgus alignment of the tibial component, which increases over-coverage, so this alignment should be avoided.

## Data Availability

A data set will be available by contacting the corresponding author.
